# Three-dimensional convolutional neural network-based classification of chronic kidney disease severity using kidney MRI

**DOI:** 10.1038/s41598-024-66814-3

**Published:** 2024-07-09

**Authors:** Keita Nagawa, Yuki Hara, Kaiji Inoue, Yosuke Yamagishi, Masahiro Koyama, Hirokazu Shimizu, Koichiro Matsuura, Iichiro Osawa, Tsutomu Inoue, Hirokazu Okada, Naoki Kobayashi, Eito Kozawa

**Affiliations:** 1 Department of Radiology, Saitama Medical University, 38 Morohongou, Moroyama-machi, Iruma-gun, Saitama, Japan; 2 Department of Nephrology, Saitama Medical University, 38 Morohongou, Moroyama-machi, Iruma-gun, Saitama, Japan; 3 School of Biomedical Engineering, Faculty of Health and Medical Care, Saitama Medical University, 38 Morohongou, Moroyama-machi, Iruma-gun, Saitama, Japan

**Keywords:** Three-dimensional convolutional neural network, Deep learning, Chronic kidney disease, Magnetic resonance imaging, Dixon-based T1-weighted image, Kidney diseases, Chronic kidney disease

## Abstract

A three-dimensional convolutional neural network model was developed to classify the severity of chronic kidney disease (CKD) using magnetic resonance imaging (MRI) Dixon-based T1-weighted in-phase (IP)/opposed-phase (OP)/water-only (WO) imaging. Seventy-three patients with severe renal dysfunction (estimated glomerular filtration rate [eGFR] < 30 mL/min/1.73 m^2^, CKD stage G4–5); 172 with moderate renal dysfunction (30 ≤ eGFR < 60 mL/min/1.73 m^2^, CKD stage G3a/b); and 76 with mild renal dysfunction (eGFR ≥ 60 mL/min/1.73 m^2^, CKD stage G1–2) participated in this study. The model was applied to the right, left, and both kidneys, as well as to each imaging method (T1-weighted IP/OP/WO images). The best performance was obtained when using bilateral kidneys and IP images, with an accuracy of 0.862 ± 0.036. The overall accuracy was better for the bilateral kidney models than for the unilateral kidney models. Our deep learning approach using kidney MRI can be applied to classify patients with CKD based on the severity of kidney disease.

## Introduction

Chronic kidney disease (CKD) is a collective term for diseases that affect the structure and function of the kidneys, and its various clinical manifestations are partially related to the cause, severity, and rate of progression. The Kidney Disease Improving Global Outcome guidelines suggest a risk-based evaluation and management of CKD and propose a CKD classification based on the cause, estimated glomerular filtration rate (eGFR) category, and albuminuria category^1^. The eGFR categories are assigned from G1 to G5. Among them, the most essential cutoff points of eGFR are 60 and 30 mL/min/1.73 m^2^ (as the borderline of G2/G3 and G3/G4, respectively). Mortality risk increased as eGFR decreased below 60 mL/min/1.73 m^2^^2^. Furthermore, for patients with an eGFR < 30, contrast media is available but cannot be administered owing to safety risks in patients with low kidney function^3^.

Magnetic resonance imaging (MRI) can provide detailed information about the internal structure of the kidney; therefore, it has been used to noninvasively assess CKD progression. Using several methods, MRI can provide data on the physiological aspects of the kidneys, including phase contrast MRI and arterial spin labeling that measure renal blood flow and perfusion, blood oxygenation level-dependent (BOLD) MRI that indirectly measures tissue oxygenation, diffusion-weighted imaging (DWI), intravoxel incoherent motion that can perceive water molecule diffusivity and capillary perfusion, and T1 and T2 mapping to detect micromolecular changes such as fibrosis and water content^4–11^. In contrast, Dixon-based gradient-echo MRI, which is routinely performed in abdominal imaging, has not been well studied in CKD evaluation; however, a recent study reported that CKD progression is associated with renal lipid accumulation quantified by Dixon-based fat fraction measurements^12^.

Recently, an emerging technique of radiomics-based machine learning and deep learning algorithms that acquire quantitative diagnostic information from medical imaging have been used for CKD diagnosis and prognosis. These novel biomarkers provide important information that cannot be easily identified by visual inspection of images with the naked eye. Previous studies have demonstrated the utility of radiomics analysis based on DWI, BOLD, T1, and T2 mapping^13,14^. Although few studies on radiomics analysis of other essential MRI sequences exist, we recently presented the feasibility of radiomics analysis of Dixon-based T1-weighted imaging (T1WI) for classifying patients with CKD based on disease severity grade^15^. We believe that Dixon-based T1WI plays an important role because it reflects the structural features of the kidneys. Another impressive use of deep learning was proposed by Kuo et al.^16^, who studied a method for estimating the eGFR using deep learning and ultrasound images. Although radiomics-based approaches have been well studied, deep learning-based classification of CKD severity using kidney MRI remains poorly understood. Therefore, testing deep learning methods is valuable.

Recent advances in artificial intelligence have been driven in part by the proliferation of comprehensive open-source deep learning resources that provide researchers with a wide variety of libraries and packages. One of these open-source libraries is FastAI^17^, which provides a rapid approximation of the outcome for deep learning models, such as the Residual Network (ResNet) model, via graphics processing unit (GPU) acceleration and a faster callback mechanism. This enables faster execution of the model with less code, and yields better precision. Furthermore, Faimed3D, an extension to FastAI, allows the implementation of a three-dimensional (3D) convolutional neural network (CNN)^18^. While 2D convolution takes 2D data and outputs a 2D result, most medical images are 3D volume data. Thus, a 3D CNN would be favorable because it performs a convolution operation in three directions. However, studies on the 3D CNN-based classification of CKD grades are limited, potentially owing to the computational cost and time required.

Therefore, this study aimed to develop a 3D CNN model to classify CKD grade using kidney MRI and the Faimed3D algorithm.

## Methods

### Participants

This study was approved by the Research Ethics Committee of the Saitama Medical University Hospital (approval number 2022-107). All experiments were performed in accordance with relevant guidelines and regulations. The requirement for informed consent was waived by the Research Ethics Committee of Saitama Medical University Hospital.

**Figure 1:**
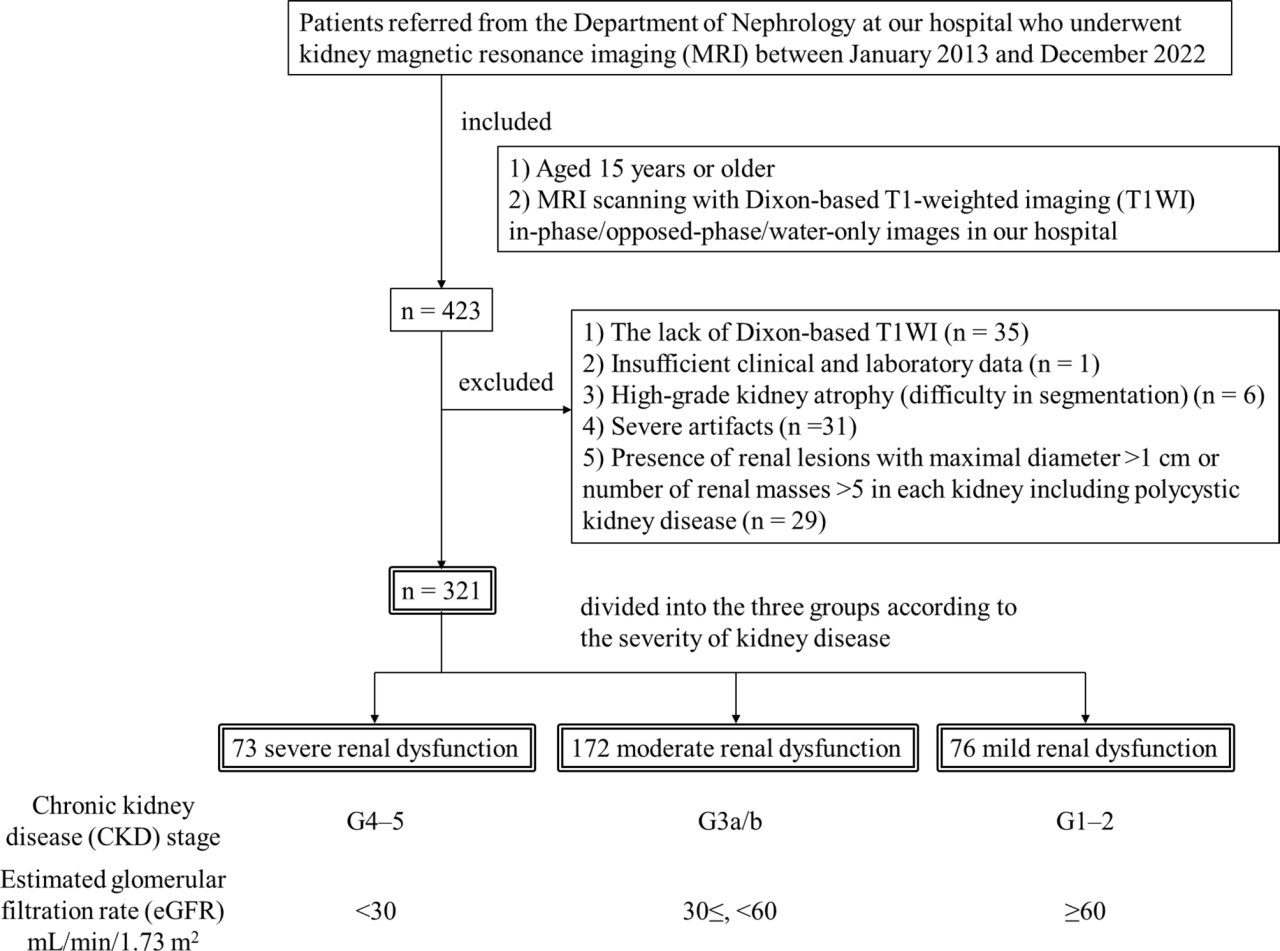
Flow chart of the inclusion and exclusion criteria for the study.

The enrolled participants partially overlapped with those in our previous work on radiomic analysis and automatic segmentation of kidney MRI, which were not relevant to the present study. Figure 1 summarizes the inclusion and exclusion criteria. We identified and reviewed 423 patients referred from the Department of Nephrology at our hospital who underwent kidney MRI between January 2013 and December 2022. The inclusion criteria included: (1) patients ≥ 15 years and (2) MRI scanning with Dixon-based T1-weighted in-phase (IP)/opposed-phase (OP)/water-only (WO) images in our hospital. The exclusion criteria included: (1) lack of Dixon-based T1WI (n = 35); (2) insufficient clinical or laboratory data (n = 1); (3) high-grade kidney atrophy (difficulty in segmentation) (n = 6); (4) severe artifacts on MRI (n = 31); and (5) presence of renal lesions with maximal diameter > 1 cm or number of renal masses > 5 in each kidney, including polycystic kidney disease (n = 29). In total, 321 patients participated in this study.

The eGFR was calculated using Eq. 1:

1 $$ {\text{eGFR}}\left( {{\text{mL}}/{\text{min}}/{1}.{73}\;{\text{m}}^{{2}} } \right) = {194} \times {\text{sCr}}^{{ - {1}.0{94}}} \times {\text{age}}^{{ - 0.{287}}} \times 0.{739}\left( {{\text{should}}\;{\text{sex}}\;{\text{be}}\;{\text{female}}} \right), $$

where age is in years and serum creatinine (sCr) is in mg/dL. The eGFR was defined as 120 mL/min/1.73 m^2^ should it be > 120 mL/min/1.73 m^2^ as calculated using Eq. 1^19^.

The patients were divided into the following three groups according to the eGFR: 73 patients with severe renal dysfunction (se-RD, eGFR < 30 mL/min/1.73 m^2^, CKD stage G4–5); 172 with moderate renal dysfunction (mo-RD, 30 ≤ eGFR < 60 mL/min/1.73 m^2^, CKD stage G3a/b); and 76 with mild renal dysfunction (mi-RD, eGFR ≥ 60 mL/min/1.73 m^2^, CKD stage G1–2).

Table 1 details the distribution of the study population in each eGFR group.

**Table 1:** Demographic and clinical characteristics of the study population.

Variable	se-RD	mo-RD	mi-RD	*P*
*N*	36	85	45	
Age, years, mean ± SD	60.9 ± 16.4	62.3 ± 13.3	43.7 ± 18.1	< 0.001
Sex, male, *n* (%)	26 (72)	57 (67)	19 (42)	0.006
Hypertension, *n* (%)	29 (81)	41 (48)	10 (22)	< 0.001
Diabetes, *n* (%)	12 (33)	11 (13)	2 (4)	< 0.001
IgA nephropathy, *n* (%)	5 (14)	10 (12)	7 (16)	0.54
Nephrotic syndrome, *n* (%)	1 (2.8)	2 (2)	2 (4.4)	0.51
eGFR, mL/min/1.73 m^2^, mean ± SD	19.8 ± 7.7	46.3 ± 8.1	78.1 ± 16.7	< 0.001

Except where otherwise indicated, data are presented as number (%) of patients. se-RD, severe renal dysfunction (eGFR < 30 mL/min/1.73 m^2^, CKD stage G4–5); mo-RD, moderate renal dysfunction (30 ≤ eGFR < 60 mL/min/1.73 m^2^, CKD stage G3a/3b); mi-RD, mild renal dysfunction (eGFR ≥ 60 mL/min/1.73 m^2^, CKD stage G1–2); IgA, immunoglobulin A; SD, standard deviation. CKD, chronic kidney disease.

### Data

MRI images were acquired using a 3.0-T superconducting unit (Skyra; Siemens Healthcare, Erlangen, Germany) with a spine coil and an 18-channel phased-array body coil. For all participants in the present analysis, we obtained Dixon-based T1-weighted IP/OP/WO images (only IP/OP/WO images were used in this analysis, as other images, including fat-only images and fat fraction ratio maps, were not generated for all patients). Representative scanning parameters for T1-weighted IP/OP/WO images were as follows: repetition time = 5.35 ms, echo time = 2.46 and 3.69 ms, flip angle = 10°, slice thickness = 3 mm, field of view = 360 × 360 × 144 mm, and recon matrix = 320.

The original image volume consisted of 40–48 2D coronal slices, each 320 × 320 pixels in size. The coronal image slice spacing was 3.0 mm and the in-plane pixel resolution was 1.125 × 1.125 mm^2^.

### Image processing and model implementation

**Figure 2:**
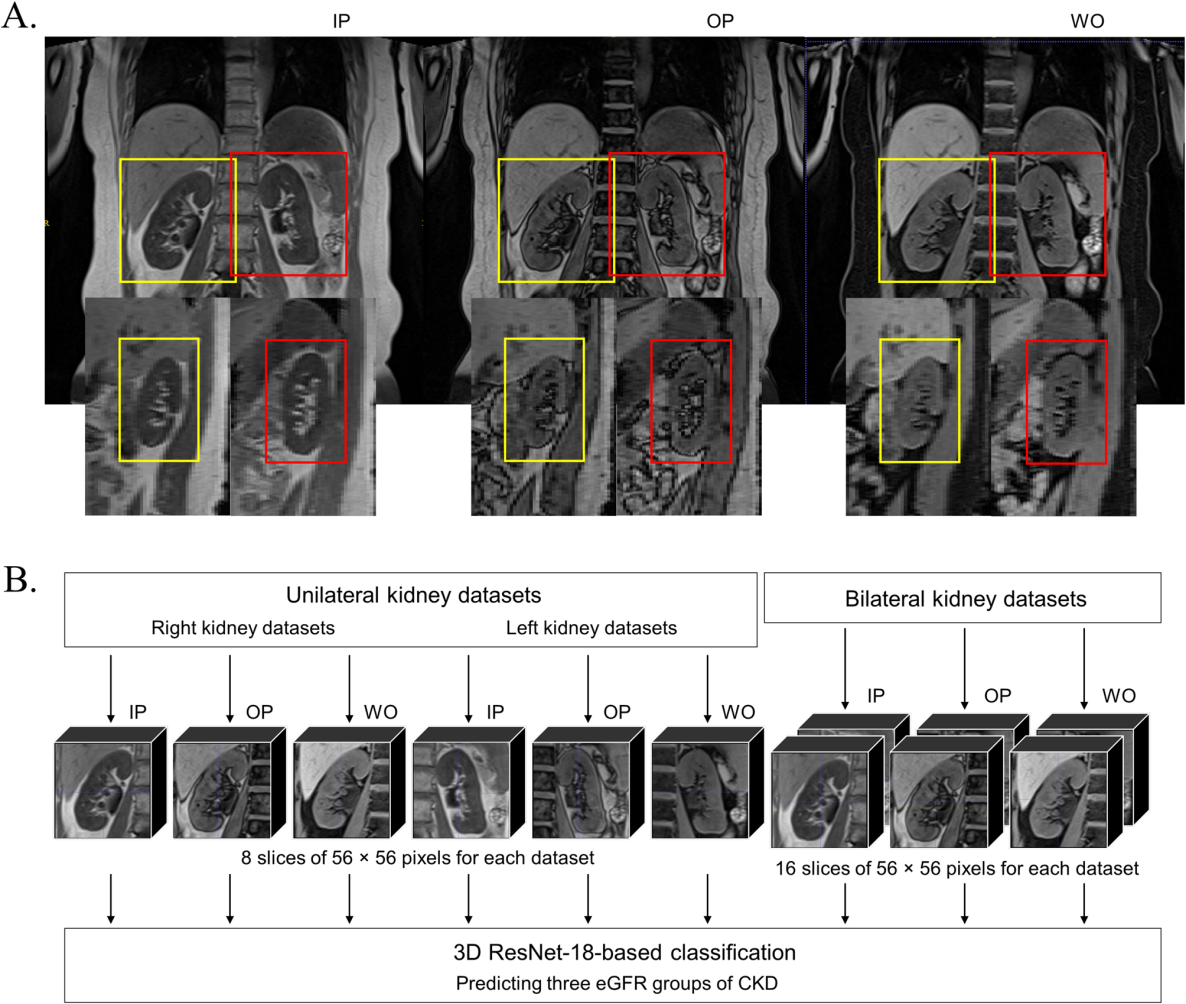
An overview of the image data processing used in this study. (**A**) On each Dixon-based T1-weighted kidney MRI, images are cropped to include the right and left kidneys; the resulting image volume has 24 coronal slices with each slice as 128 × 128 px. (**B**) These images are then further resized into 8 slices of 56 × 56 px for unilateral kidney datasets. Bilateral kidney data are obtained by stacking the data of the right and left kidneys, hence 16 slices of 56 × 56 px. Therefore, a total of 9 datasets are created for three-dimensional (3D) convolutional neural network (CNN) models derived (1) from each unilateral kidney (right or left kidney) and for bilateral kidneys, and (2) from each imaging method (T1-weighted in-phase (IP)/opposed-phase (OP)/water-only (WO) images), respectively. The 3D residual network-18 (3D ResNet-18)-based classification is performed on each dataset, classifying the three severity groups of chronic kidney disease (CKD).

We constructed 3D CNN models for the bilateral kidneys, each unilateral kidney (right or left), and each imaging method (T1-weighted IP/OP/WO images). We evaluated and compared their classification performances. An overview of the image data processing scheme is shown in Fig. 2.

Image preprocessing was performed using open-source software (3D slicer version 5.0.3). For each kidney MRI, images were cropped to include the right and left kidneys; the resulting image volume had 24 coronal slices with each slice of 128 × 128 px. Bilateral kidney data were obtained by stacking the data of right and left kidneys; hence, 48 slices of 128 × 128 px were obtained. These images were then further resized into 8 slices of 56 × 56 px for unilateral kidney data, and 16 slices of 56 × 56 px for bilateral kidney data. The voxel intensity distribution of each image was normalized by rescaling the intensities into the range of [0, 255].

All image data were subsequently converted into standard binary files (.npy) format and saved together in one directory, along with a CSV file containing clinical information such as eGFR groups. The data were randomly split into 70% for training, 10% for validation, and 20% for testing. We performed five-fold cross-validation, each with 20% of the available data for the test. This enabled the models to be tested on the entire dataset.

The 3D CNN model was trained using Python 3.9 (Python Software Foundation, Beaverton, OR, USA) and the Faimed3D library (https://kbressem.github.io/faimed3d) with Pytorch 1.9.0 (Facebook’s AI Research lab) backend. Data transformation and augmentation were performed using Faimed3D transformations: randomly flipping the input image along any axis in 3D; randomly rotating the input image by 90° (or 180°and 270°) at an arbitrary angle; randomly cropping the 3D volume; randomly adjusting the brightness and contrast; and randomly generating various artifact imitations with warping, sheering, trapezoid, Gaussian noise, and blur.

**Figure 3:**
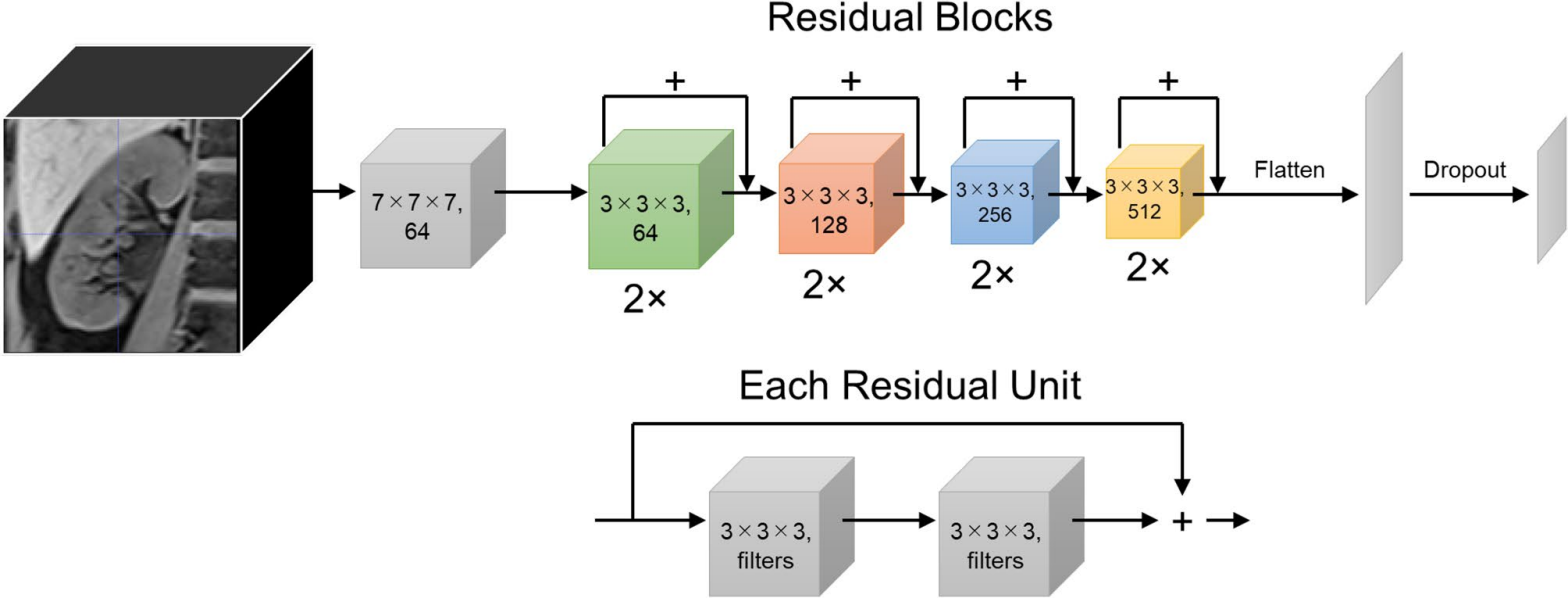
The architecture of our three-dimensional residual network-18 model. Input is processed volumetric data of kidney magnetic resonance imaging. The network contains the initial convolutional layer, followed by 8 residual units (two with filters = 64, two with filters = 128, two with filters = 256, and two with filters = 512), each with two convolutional blocks as shown in the bottom row. The last layer is a fully connected dense layer that outputs a classification of three groups.

Our 3D CNN model is based on the 3D ResNet architecture included in the Faimed3D software package. We utilized the default ResNet 3D-18 network, which was pre-trained on the action recognition dataset. Figure 3 shows the architecture of ResNet 3D-18. The models were trained for 100 iterations on a Windows 10 workstation with a single GeForce RTX 3090 Graphics Processing Unit. Overall, five models were trained (one per fold), with a training time of approximately 10 min per fold. The trained model was subsequently used to perform classification of the test data for approximately 5 s. Finally, the model predictions were averaged and compared with the basic classification metrics, including accuracy, precision, recall/sensitivity, specificity, and f1 score.

The accuracy was calculated using Eq. 2:

2 $$ Accuracy\left( {y,\hat{y}} \right) = \frac{1}{{n_{samples} }}\mathop \sum \limits_{i = 0}^{{n_{samples} - 1}} 1\left( {\hat{y}_{i} = y_{i} } \right) $$

where $\hat{y}_{i}$ is the predicted value of the *i*-th sample, $y_{i}$ is the corresponding true value, and $n_{samples}$ is the number of samples.

The precision, recall/sensitivity, specificity and f1 score were calculated using Eq. 3:

3 $$ \begin{aligned} Precision & = TP/\left( {TP + FP} \right) \\ Recall/sensitivity & = TP/\left( {TP + FN} \right) \\ Specificity & = TN/\left( {TN + FP} \right) \\ F1 score & = 2 \times TP/\left( {2 \times TP + FP + FN} \right) \\ \end{aligned} $$

where *TN*, TP, FN, and FP are the numbers of true negatives, *TP* is the number of true positives, *FN* is the number of false negatives, and *FP* is the number of false positives.

A receiver operating characteristic (ROC) curve was generated by plotting the true positive rate (TPR) against the false positive rate (FPR) at various threshold settings. The TPR is also known as sensitivity, and the FPR is one minus the specificity. ROC-AUC computes the area under the ROC curve.

The macro-averaged and weighted scores were calculated using Eq. 4:

4 $$ \begin{aligned} Macro\;score & = \frac{{\mathop \sum \nolimits_{i = 1}^{N} Score_{i} }}{N} \\ Weighted\;score & = \mathop \sum \limits_{i = 1}^{N} w_{i} \times Score_{i} \\ \end{aligned} $$

where *N* is the number of classes and *w*_*i*_ is the number of samples in class *i* divided by the total number of samples.

Furthermore, we calculated the Matthews correlation coefficient (MCC) for multi-class classification using a goodness of fit test.

The MCC was calculated using Eq. 5:

5 $$ MCC = \frac{{c \times s - \mathop \sum \nolimits_{k}^{K} p_{k} \times t_{k} }}{{\sqrt {\left( {s^{2} - \mathop \sum \nolimits_{k}^{K} p_{k}^{2} } \right) \times \left( {s^{2} - \mathop \sum \nolimits_{k}^{K} t_{k}^{2} } \right)} }} $$

where *c* is the number of correctly predicted samples, *K* is the total number of classes, *k* is the class from 1 to *K*, *s* is the number of samples, *t*_*k*_ is the number of times class *k* truly occurs, *p*_*k*_ is the number of times class *k* is predicted.

All statistical analyses were performed using the open-source software package (Python sci-kit-learn 0.22.1)^20^. Statistical significance was set at *P* < 0.05.

## Results

**Figure 4:**
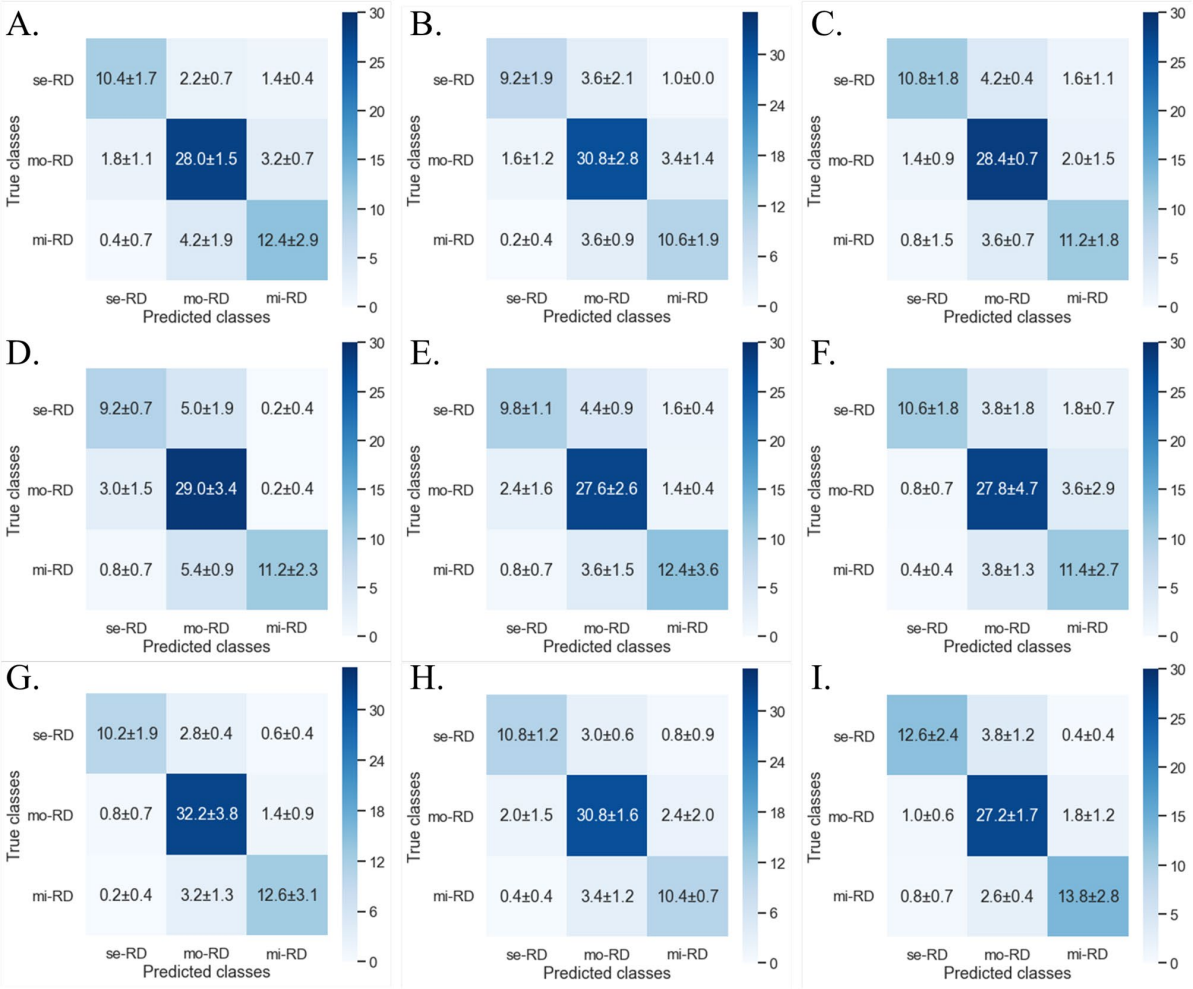
Confusion matrices show the status of multi-class classifications using bilateral (**A**–**C**), right (**D**–**F**) and left (**G**–**I**) kidney datasets with Dixon-based T1-weighted in-phase image (**A**, **D**, **G**), opposed-phase image (**B**, **E**, **H**), and water-only image (**C**, **F**, **I**), in classifying the three groups of chronic kidney disease. se-RD: severe renal dysfunction (estimated glomerular filtration rate [eGFR] < 30 mL/min/1.73 m^2^), mo-RD: moderate renal dysfunction (eGFR ≥ 30 and < 60 mL/min/1.73 m^2^), and mi-RD: mild renal dysfunction (eGFR ≥ 60 mL/min/1.73 m^2^). The data are means ± standard deviations.

The findings of the classification models are summarized in Table 2. Figure 4 shows the confusion matrices of all the classification attempts. The best performance was obtained when using an IP image of bilateral kidneys, with an accuracy of 0.862 ± 0.036. Compared with the models for the right or left kidney, those for bilateral kidneys afforded better classification performance. Among the models for bilateral kidneys, the OP images provided inferior results, whereas the IP images provided superior performance. Among the models for the right or left kidney, no differences were noted between the T1WI IP/OP/WO images.

**Table 2:** Performance of three-dimensional convolutional neural network-based classification of the three groups of chronic kidney disease.

	Accuracy	Precision	Recall/sensitivity	Specificity	F1 score	ROC AUC	MCC
Bilateral kidneys							
IP	0.862 ± 0.036	0.878 ± 0.043	0.818 ± 0.037	0.911 ± 0.020	0.842 ± 0.040	0.936 ± 0.020	0.601 ± 0.047
		0.866 ± 0.037	0.859 ± 0.036	0.873 ± 0.031	0.857 ± 0.036	0.937 ± 0.016	
OP	0.812 ± 0.045	0.816 ± 0.069	0.790 ± 0.036	0.889 ± 0.023	0.792 ± 0.053	0.916 ± 0.019	0.523 ± 0.119
		0.825 ± 0.053	0.813 ± 0.045	0.854 ± 0.027	0.812 ± 0.045	0.916 ± 0.018	
WO	0.836 ± 0.031	0.852 ± 0.047	0.816 ± 0.032	0.909 ± 0.016	0.826 ± 0.034	0.928 ± 0.032	0.583 ± 0.106
		0.846 ± 0.037	0.838 ± 0.032	0.888 ± 0.021	0.836 ± 0.032	0.923 ± 0.028	
Right kidney							
IP	0.774 ± 0.029	0.808 ± 0.032	0.732 ± 0.026	0.864 ± 0.010	0.752 ± 0.020	0.880 ± 0.024	0.402 ± 0.038
		0.801 ± 0.021	0.772 ± 0.029	0.819 ± 0.021	0.769 ± 0.027	0.877 ± 0.017	
OP	0.778 ± 0.025	0.776 ± 0.039	0.742 ± 0.041	0.874 ± 0.019	0.750 ± 0.037	0.862 ± 0.010	0.391 ± 0.068
		0.782 ± 0.028	0.778 ± 0.025	0.843 ± 0.034	0.773 ± 0.028	0.858 ± 0.014	
WO	0.778 ± 0.035	0.800 ± 0.036	0.742 ± 0.025	0.864 ± 0.010	0.756 ± 0.026	0.880 ± 0.024	0.397 ± 0.014
		0.801 ± 0.021	0.772 ± 0.029	0.819 ± 0.021	0.769 ± 0.027	0.877 ± 0.017	
Left kidney							
IP	0.796 ± 0.045	0.794 ± 0.058	0.774 ± 0.038	0.884 ± 0.024	0.778 ± 0.042	0.884 ± 0.021	0.404 ± 0.024
		0.799 ± 0.045	0.794 ± 0.043	0.859 ± 0.029	0.793 ± 0.043	0.878 ± 0.022	
OP	0.792 ± 0.015	0.790 ± 0.024	0.758 ± 0.044	0.873 ± 0.017	0.762 ± 0.026	0.878 ± 0.027	0.397 ± 0.042
		0.799 ± 0.020	0.791 ± 0.016	0.828 ± 0.041	0.788 ± 0.018	0.882 ± 0.024	
WO	0.802 ± 0.029	0.822 ± 0.047	0.774 ± 0.045	0.888 ± 0.018	0.784 ± 0.045	0.880 ± 0.017	0.395 ± 0.052
		0.819 ± 0.035	0.800 ± 0.032	0.864 ± 0.026	0.799 ± 0.033	0.874 ± 0.020	

Data are presented as means ± standard deviation. IP, in-phase; OP, opposed-phase; WO, water-only. MCC, Matthews’ correlation coefficient.

## Discussion

In this study, we developed a 3D CNN model to classify CKD severity using T1-weighted IP/OP/WO kidney MRIs. Our results showed that the Faimed3D framework with 3D ResNet architecture successfully classified patients with CKD based on the eGFR grade. The best overall accuracy was observed for the model constructed from the IP image of the bilateral kidneys, whereas inferior results were obtained for the other images and unilateral kidney models.

Machine learning and deep learning-based analyses of kidney MRI have been shown to be feasible for classifying CKD severity^13–16^. Most studies have examined machine learning methods using MRI-based radiomics analysis to assess the renal function in patients with CKD. Notably, our group previously reported that a radiomics model based on Dixon-based T1WI provided adequate classification accuracy for CKD grades^15^. In the present study, Dixon-based T1WI was investigated in combination with a deep learning approach, and the classification performance seemed to improve compared to previous results, probably owing to the convolutional network used in this study. In a previous radiomics analysis, WO images performed better than IP and OP images. Intriguingly, in this study, IP images performed the best, followed by WO and OP images.

Several aspects of the results are worth discussing. Dixon-based techniques, also called chemical shift imaging, deal with the different phase (IP and OP) cycling of fat and water, and allow the acquired images to be computed into four sequences: IP/OP/WO/fat-only (FO) images^21^. The T1-weighted IP and WO images correspond to “nearly” non-fat-suppressed and fat-suppressed T1WI, respectively, whereas the OP image is an intermediate image that shows micro-fat with low signal intensity. In this regard, our 3D CNN model exhibited the best performance with IP images, which may indicate that non-fat-suppressed T1WI are more suited to our model than fat-suppressed images. Notably, a recent study on T1 mapping showed that increased cortical T1 values and decreased T1 corticomedullary differentiation were associated with the severity of renal impairment^22,23^. Changes in T1 values may be ascribed to renal physiological conditions such as hypoxia, fibrosis, and inflammation^22,24–27^. Our hypothesis is that these changes in T1 values are represented on Dixon-based T1WIs, especially IP images, and may provide clues for CNN to classify the CKD grade.

Additionally, Dixon-based images have been used for homogeneous fat suppression or fat quantification and provide better signal-to-noise efficiency than other conventional fat-suppression methods^28^. In diabetic nephropathy, lipid deposition in the kidneys can be measured using Dixon-based techniques^29^. In recent studies, Dixon-based fat quantification has shown the potential for discriminating the severity of CKD. Notably, it has been suggested that lipid accumulation occurs in the renal parenchyma, especially in the cortex, and that this accumulation increases in proportion with the CKD grade^12^. Furthermore, in Dixon-based imaging, double-echo sequences have been reported to detect iron deposition and magnetic susceptibility artifacts associated with T2* effects^30^. In this context, the renal signal changes caused by lipid or iron accumulation may have helped our 3D CNN model classify CKD grades. This hypothesis can be further verified if quantitative images, such as fat fraction maps, are introduced into our 3D CNN models.

In a study on automated kidney segmentation of Dixon-based T1WI, the model created using IP images showed the highest segmentation accuracy, followed by those created using WO and OP images^31^. Among Dixon-based T1WI, IP images may have favorable image contrast for the CNN to identify the true renal parenchyma compared with other images^31^. This observation stems from the idea that the location of the kidney relative to the adjacent liver and spleen, and the contrast between the kidney and these organs, could provide clues for CNN to identify the renal parenchyma. Notably, signal contrast with the surrounding adipose tissue may contribute to kidney identification. Moreover, the internal contrast of the kidney and the contrast difference between the kidneys could help identify features for classifying the grade of renal dysfunction. To test the above hypothesis, class activation mapping, which shows where the CNN is focused, could be useful. Comparison of the performance of the CNN model created with the images used in this study and kidney-only images with the surrounding tissue removed using a mask may also be useful for determining the influence of the surrounding tissue. This aspect will be investigated in future studies.

Our study showed that the classification performance was better for the bilateral kidney models than for the unilateral kidney models. Previous studies on MRI-based CKD classification using machine learning or deep learning have not considered whether one or both sides of the kidney should be evaluated. Most studies tend to consider only one side of the kidney for several reasons. Severe artifacts tend to occur on one side (especially the left side)^13^, and for radiomics analysis, evaluation of only one side can reduce the time-consuming process of region-of-interest delineation^15^. However, as the present study suggests, the imaging data of bilateral kidneys might contain more integrated and beneficial information for renal function than the data of unilateral kidneys.

This pilot study investigated the applicability of Faimed3D-based CNN models for classifying CKD severity using kidney MRI. Faimed3D is a recently released open-source library that allows the implementation of 3D CNN models on radiological data^18^. Although the model is not beyond the state-of-the-art, Faimed3D emphasizes usability and speed^18^. 3D CNNs have disadvantages in terms of computational cost and time required; however, they have been successfully applied in several recent studies. In the Faimed3D framework, GPU acceleration and a faster callback mechanism can be used to accelerate training and validation with less code and yield better precision^18^. Therefore, we completed the training and validation processes in approximately 10 min per fold. Most medical images consist of 3D volumetric data; therefore, 3D CNNs are favorable because they perform convolution operations in three directions. In 3D networks, the image volume is divided into smaller cubes to allow different input shapes, thus reducing memory requirements^32^. In other aspects, the 3D CNN facilitates data integration, such as when evaluating bilateral kidneys, as in the present study.

Although machine learning-aided radiomics analysis has been well studied, deep convolutional networks using medical images have not been fully tested in the assessment of CKD status. CNN-based studies on CKD diagnosis and prognosis have primarily focused on clinical and serological datasets^33–35^. This may be because kidney MRI is not a routine examination for CKD in a clinical setting. Most of these studies were non-image-based, and the overall performance of the models was excellent, with accuracy scores > 90%^33,34,36^. All these non-image-based studies achieved good accuracy with binary classification, making exact comparisons between their studies and ours difficult. Considering the good performance of the CNN models based on clinical and laboratory data, it may be possible to develop more sophisticated models by integrating images with clinical and laboratory data in the future. This is a preliminary study to evaluate the performance of a 3D CNN model that simply classifies CKD grades using kidney MRI. However, our future goal is to develop alternative imaging-based biomarkers that cannot be identified using existing methods. In this context, a recent study using an MRI-based CNN to predict the eGFR decline over time in patients with autosomal dominant polycystic kidney disease is intriguing^37^. Therefore, image-based models (and non-image-based models) for renal function assessment should be integrated to generate a more comprehensive and meaningful model for predicting CKD progression and eGFR decline. Further research is required to confirm the validity and generalizability of these models.

This study has several limitations. First, this retrospective study enrolled 321 patients from a single institution; this was a small sample size for a deep learning-based study with some imbalance between each CKD group. Future studies should examine a larger number of patients with a more balanced grouping. Second, because we excluded patients with renal lesions, some important renal diseases, such as polycystic kidney disease, were excluded from the analysis. Third, we could not analyze other Dixon-based images such as fat-only images and fat fraction ratio maps because they were not available for all patients. Fourth, other T1- or T2-weighted images were not available for use in this study because they were not routinely scanned or scanned in different planes in routine sequences.

In conclusion, a 3D CNN model was developed to classify CKD severity using T1-weighted IP/OP/WO MRIs. The Faimed3D framework with the 3D ResNet architecture can be successfully applied to classify patients with CKD according to disease severity. The overall accuracy was better for the bilateral kidney models than for the unilateral kidney models. The best performance was observed for the model created with an IP image of bilateral kidneys, whereas inferior results were obtained for other images and unilateral kidney models. As our preliminary 3D CNN model can be extended to be more comprehensive and meaningful, further validation of these results is required in the future.

## Data Availability

The datasets used and/or analyzed in the current study are available from the corresponding author upon reasonable request.

## Abbreviations

3D: Three-dimensional
ADC: Apparent diffusion coefficient
BOLD: Blood oxygen level-dependent imaging
CKD: Chronic kidney disease
CNN: Convolutional neural network
DWI: Diffusion-weighted imaging
eGFR: Estimated glomerular filtration rate
IP: In-phase
MRI: Magnetic resonance imaging
OP: Opposed-phase
ResNet: Residual network
T1WI: T1-weighted imaging
WO: Water-only
